# Development of inequalities in fruit and vegetable intake through early adulthood: insights from household panel surveys in the United Kingdom and Australia

**DOI:** 10.1038/s41430-025-01609-x

**Published:** 2025-04-03

**Authors:** Tanya Braune, Katherine M. Livingstone, Jean Adams, Eleanor M. Winpenny

**Affiliations:** 1 MRC Epidemiology Unit, Level 3 Institute of Metabolic Science, University of Cambridge School of Clinical Medicine, Cambridge, CB2 0SL UK; 2 Institute for Physical Activity and Nutrition (IPAN), School of Exercise and Nutrition Sciences, Deakin University, 3220 Geelong, VIC Australia; 3 Mohn Centre for Children’s Health and Wellbeing, School of Public Health, Imperial College London, London, UK

**Keywords:** Risk factors, Epidemiology

## Abstract

**Background:**

Adequate fruit and vegetable intake is important for preventing chronic disease. The transition from adolescence to early adulthood involves changes in their social and environmental context, lifestyle and behaviours that may determine lifelong dietary patterns. Differences that emerge between populations over this age range may lead to long-term health inequalities. This study examined the trajectories of fruit and vegetable intake among young adults in the United Kingdom (UK) and Australia.

**Methods:**

We analysed data from young adults aged 16–30 years from the UK Household Longitudinal Study (UKHLS; *n* = 2751) and the Household, Income and Labour Dynamics in Australia Survey (HILDA; *n* = 6255). Hybrid mixed-effect regression models were fitted to assess intake trajectories of fruit and vegetables, exploring differences by sex and socioeconomic position.

**Results:**

Fruit intake decreased and vegetable intake increased with age in both countries. Fruit intake trajectories differed by sex in both cohorts, while vegetable intake trajectories differed by sex in Australia only. Parental education was a predictor of fruit and vegetable intake trajectories in both cohorts, with differences between groups with high and low parental education narrowing with age in Australia for fruit intake.

**Conclusions:**

The differing trajectories emphasise the importance of understanding contextual influences to effectively tailor public health strategies. Our study highlights early adulthood as a critical period for developing dietary patterns that could influence long-term health outcomes, with differences between groups emerging quickly. Addressing these inequalities is essential when developing policies aimed at improving diet among young adults and reducing the prevalence of chronic disease.

## Introduction

Fruit and vegetables are major components of a healthy diet, and their intake has been associated with a lower relative risk of chronic disease in meta-analyses, including cardiovascular disease [1] and type-2 diabetes mellitus [2]. Obesity, characterised by elevated body mass index (BMI), is among the top risk factors for disease globally, estimated to be responsible for 3.4 million deaths in 2010 [3]. A systematic analysis of 1769 global studies demonstrated that the prevalence of overweight and obesity has grown worldwide, increasing by 47.1% between 1980 and 2013 [4]. A population-based analysis of over 2 million adults in England found that young adults (18–24 years) are at the highest risk of weight gain and increased BMI compared to older adults [5]. Over a 10-year period, they face a 37% greater absolute risk of moving from normal weight to overweight or obesity, and a 42% higher absolute risk of progressing from overweight to obesity [5], highlighting the importance of understanding weight-related dietary behaviours in this age group. Adequate fruit and vegetable intake is particularly important during adolescence and early adulthood in contributing to overall diet quality and risk of onset of overweight and obesity, which could continue in adulthood, contributing to future chronic disease [6–8].

The transition from late adolescence to early adulthood is a period of change, characterised by increased independence and the establishment of lifestyle habits, such as dietary patterns [9]. Despite the importance of dietary patterns in this age group, there have been few studies covering this longitudinally, limiting our understanding of the nuances across this transitional period [10]. In the present study, the age group of interest includes individuals aged 16–30 years, to capture this period of early adulthood [11] and we refer to this popuplation as ‘young adults’. This period is particularly crucial for examining trajectories in fruit and vegetable intake as it encompasses a time when individuals often experience changes in living situations, educational environments, and employment status, all of which can influence dietary patterns and habits.

The UK and Australia share social and cultural overlaps, for example, access to healthcare through their universal healthcare systems [12, 13], as well as language, political and economic similarities [14, 15]. Both of these countries face comparable health challenges including overweight and obesity rates around 65% [16] for individuals aged 15 years and older, and difficulties in meeting fruit and vegetable consumption guidelines, despite public health efforts [17–19]. The World Health Organization (WHO) advises a minimum intake of 400 g or five portions per day of fruits and vegetables [20, 21], informing the UK’s 5-a-day recommendation [22]. The National Health and Medical Research Council (NHMRC) 2013 Australian Dietary Guidelines build on this, recommending a minimum of two portions of fruit and five portions of vegetables per day [23], also known as the ‘2&5 campaign’. Despite minor variations in the recommendations, the overarching goal remains the same: to boost the intake of fruits and vegetables. Whilst there are a number of similarities between the UK and Australia, there may be contextual factors that drive fruit and vegetable intake, such as cost, availability, family food culture and school food policies [24]. Taking a comparative approach across both countries, as we do in this paper, not only facilities a broader understanding of dietary trends in this age group, but also aids in the identification of universally applicable interventions as well as country-specific strategies.

Previous dietary trajectory studies in Australia [25], Norway [26] and the United States [27], each analysing over 1000 participants, have underscored the significance of late adolescence and early adulthood as a period of dietary and life transitions. These longitudinal studies collectively highlight the role of healthy diets during adolescence in setting the stage for long-term dietary habits. Notably, the evidence suggests that a poorer dietary pattern established during this period tends to persist or worsen as individuals enter adulthood, with male participants shown to be especially prone to experiencing a deterioration in diet quality [25]. Life events, such as moving out of the family home, was identified as a contributor to a decline in fruit and vegetable intake frequency of about 0.5 times per week [26], while beginning full-time employment and becoming a parent were associated with an increase in fast food intake frequency by 0.16 times per week [27]. Overall, these studies underscore the concerning trend that many individuals struggle to maintain a healthy diet through early adulthood.

Early adulthood marks a period for the establishment of adult dietary patterns as well as the potential emergence of dietary inequalities. Inequalities refer to differences between groups within a society, such as socioeconomic status, that are “systemic, unfair and avoidable”, as defined by the National Institute for Health and Care Excellence (NICE) [28]. In this context, the inequalities observed measure the differences in health behaviours associated with dietary intake between female and male participants and individuals with parents with a higher or lower educational background. Evidence suggests early adutlhood may be particularly pivotal in the development of diverging dietary patterns between female and male individuals [29], with female participants typically consuming more fruits and vegetables than their male counterparts [17, 30–32]. These differences may be attributed to varying health beliefs, knowledge about nutrition, and societal norms. Moreover, socioeconomic factors, including parental education and household income, play a significant role in shaping dietary behaviours [32–38]. Typically, a higher parental socioeconomic position (SEP) is associated with greater access to a variety of healthy foods, increased nutritional knowledge, and a higher likelihood of adhering to dietary guidelines. These are likely to lead to differential trends in intake between SEPs and contribute to the known inequalities in related health outcomes. Understanding these influences on dietary trajectories could help identify where public health efforts and interventions can be most effective, thus highlighting the need to focus on this age range to better understand the dietary trajectories and the roots of health disparities.

Building on these findings, our study uses nationally representative, longitudinal household panel data from the UK and Australia to analyse the dietary trajectories of young adults. The key objective of our study was to provide a nuanced analysis of how fruit and vegetable intake changes across early adulthood, considering the influence of sex and SEP. Analysis of multiple timepoints of panel data allows us to distinguish within-individual from cohort-level dietary changes, for a deeper understanding of diet trajectories during this critical period of development. Including both UK and Australian data allowed us to explore which findings may be replicable across contexts and begin to understand which aspects of each context may be driving intake. These findings could, in turn, help identify key moments for future interventions and which subgroups face the most pronounced dietary inequalities, thereby contributing to the reduction of chronic disease risk and addressing public health priorities.

Our objective was achieved through answering the following research questions:

1. What are the trajectories of fruit and vegetable intakes, across early adulthood (16–30 years), in UK and Australian populations?
2. How do these trajectories differ by sex and SEP?

## Methods

### Study design and population

Data for this analysis were drawn from the UK Household Longitudinal Study (UKHLS) [39] and the Household, Income and Labour Dynamics in Australia (HILDA) Survey [40], both up-to-date household-based nationally representative panel surveys that have collected data in the UK since 2009 and Australia since 2001. In UKHLS, households were selected to participate in the general population sample based on a proportionately stratified, equal probability, randomly selected sample of residential addresses in the UK. In HILDA, households were included using a multi-staged approach, first selecting Census Collection Districts, followed by dwellings within those based on expected response and occupancy, and finally up to three households per dwelling were selected to participate. Further details on the UKHLS and HILDA sampling and survey methods can be found elsewhere [39, 41–43].

In UKHLS, data for fruit and vegetable intake for adult household members aged 16 years and older were collected in waves 7 (2015–2016), 9 (2017–2019), 11 (2019–2021) and 13 (2021–2023). In HILDA, fruit and vegetable intake for adult household members aged 15 years and older were collected in waves 7 (2007), 9 (2009), 13 (2013), 17 (2017) and 21 (2021). This analysis included young adults aged 16–30 years at the time of data collection in any wave in both cohorts.

### Survey and measures

#### Exposures: age and sociodemographic groupings

Age, in completed years, was derived based on reported date of birth and interview date. To explore differences in sociodemographic groups, sex and parental education were explored. Sex was reported by a household member and checked for consistency across all previous waves by UKHLS during data collection.

Due to the household-based sampling method, parental education was self-reported by parents and updated at every wave to include their most recent qualification. “Parents” were defined as biological, adoptive or step-parents. In the present study, the highest achieved educational qualification of parents at the baseline wave (wave 7) was linked back to the young adults through cross-wave identifiers. If data from more than one parent was available, only the highest qualification was retained to derive a single variable. A binary variable was created for parental education from the six possible answers (degree, other higher degree, A-level etc, GCSE etc, Other, None) in UKHLS and eight possible answers in HILDA (postgrad–masters or doctorate, grad diploma or grad certificate, bachelor or honours, advanced diploma or diploma, cert III or IV, Year 12, Year 11 and below) to include 1) degree level and above and 2) no degree (any other qualification below degree level, including no qualification), dividing the populations into roughly equal groups.

#### Outcome: young adult fruit and vegetable intake (portions per day)

In both datasets, the individual level questionnaire included self-reported intake of fruit and vegetable frequency (days/week) and amount (portions/day) (described in Table 1). The HILDA Survey Team designed the questions on fruit and vegetable intake frequency and the questions on fruit and vegetable intake amount were derived from the Australian Bureau of Statistics 2004/2005 National Health Survey [43]. In UKHLS, a numerical average value was assigned to each frequency category as follows: never = 0 days, 1–3 days = 2 days, 4–6 days = 5 days, and every day = 7 days. In HILDA, the reported frequency was retained. Frequency was multiplied by the reported amount (in portions) to obtain portions per week of fruit and vegetables. Portions per day were calculated by dividing this value by seven.

**Table 1:** Dietary survey measures and calculated variables used in UKHLS and HILDA.

Measure	UKHLS	Response options^a^	HILDA	Response options
Fruit intake frequency	Including tinned, frozen, dried and fresh fruit, on how many days in a usual week do you eat fruit?	Never	Including tinned, frozen, dried and fresh fruit, on how many days in a usual week do you eat fruit?	Do not eat fruit in a usual week
		1–3 Days		1, 2, 3, 4, 5, 6 or 7 days per week
		4–6 Days		
		Every day		
Fruit amount	On the days when you eat fruit, how many portions (e.g. an apple, an orange, some grapes) do you eat?	Whole numbers	On a day when you eat fruit, how many serves of fruit do you usually eat?	1 serve
				2 serves
				3 serves
				4 serves
				5 serves
				6 or more serves
Vegetable intake frequency	Including tinned, frozen and fresh vegetables, on how many days in a usual week do you eat vegetables? Do not include potatoes, crisps or chips.	Never	Including tinned, frozen and fresh vegetables, on how many days in a usual week do you eat vegetables?	Do not eat vegetables in a usual week (0 days)
		1–3 Days		1, 2, 3, 4, 5, 6 or 7 days per week
		4–6 Days		
		Every day		
Vegetable amount	On the days when you eat vegetables, how many portions (i.e. 3 heaped tablespoons) do you eat? Please do not include potatoes.	Whole numbers	On a day when you eat vegetables, how many serves of vegetables do you usually eat?	1 serve
				2 serves
				3 serves
				4 serves
				5 serves
				6 or more serves

^a^UKHLS responses re-coded for analysis as follows:

Never = 0 days; 1-3 days = 2 days; 4-6 days = 5 days, everyday = 7 days.

^1^ United Kingdom Household Longitudinal Survey.

^2^ Household, Income and Labour Dynamics in Australia.

### Statistical methods

R statistical software [44] (2023.12.1 + 402) was the primary software used for statistical analysis. Python 3 was used for data pre-processing [45]. Descriptive statistics were computed for outcomes (fruit or vegetable intake) and exposure variables (age, sex, and parental education), using frequency/percentage and mean/standard deviation, as appropriate (see Table 2).

**Table 2:** Characteristics for UKHLS (*n* = 2751) and HILDA (*n* = 6255) weighted samples.

	*n* (%) / mean ± SD		*n* (%) / mean ± SD	
	UKHLS	*n*	HILDA	*n*
Fruit intake (portions per day)	1.42 ± 1.29	2751	1.32 ± 1.08	6255
Vegetable intake (portions per day)	1.77 ± 1.29	2751	1.98 ± 1.22	6255
Age	20.3 ± 4.67	2751	20.89 ± 4.21	6255
**Sex**				
Female	1632 (59)	2751	3186 (51)	6255
Male	1119 (41)	2751	3069 (49)	6255
**Parental highest qualification**				
Degree	1366 (55)	2489	1500 (35)	4323
No degree	1123 (45)	2489	2823 (65)	4323

#### Multilevel models

Data from the UK and Australia were analysed separately as multilevel linear regression models with measurement waves nested within individuals. Chi-squared Wald tests were run for all models to test whether the within-person effects of age differed significantly from the between-person effects. As these were shown to be significantly different, hybrid mixed effects models were used to model the relationship between both fruit and vegetable intakes separately and age, disaggregating overall effect of age into within- and between-person effects. Our analysis focusses on within-person effects to answer the research questions on trajectories of dietary intake.

We tested a non-linear term (age^2^) for the relationship but this was not significant and did not improve model fit, so we assumed a linear relationship between age and both fruit and vegetable intake. In addition, we tested the use of robust standard errors because of heteroscedasticity found in the datasets, however, these did not improve the models. We proceeded without robust standard errors as linear mixed-effects models have been shown to be robust to violation of distributional assumptions [46].

For each dataset, Models 1a and 1b looked at the association between age and daily fruit or vegetable intake, respectively. Models 2a and 2b built on Models 1a and 1b to additionally include sex and its interaction with age to investigate whether there are differences in trajectories of intake between female and male participants. Similarly, Models 3a and 3b built on Models 1a and 1b to include parental education and its interaction with age to investigate whether there were differences in trajectories of intake of fruit and vegetables between individuals with parents with a degree versus those with no degree. In our plots of predicted intake trajectories based on these models, to make the x-axis more interpretable, we have added the mean age of the overall cohort to person-centred age, so that these graphs reflect the age range of interest. Person-centred age of zero = 20.3 years in UKHLS and 20.9 years in HILDA.

All models were fitted using the *lme4* package in the R statistical environment [47]. Confidence intervals at 95% were computed using the *confint.merMod* method [48] for multilevel models. The original survey weights (see below for details) were rescaled using the *rescale_weights* method from the *‘datawizard’* package [49], allowing for the grouping structure to be accounted for when applying to survey weights to all models.

#### Missing values and outliers

We included young adult participants aged 16–30 years in UKHLS (*n* = 2751) and HILDA (n = 6255), that appeared in all waves up to and including Wave 13 (UKHLS) and Wave 21 (HILDA). Outliers were assessed and data removed for values that were greater than three times the interquartile range of intake for fruit or vegetables. This was done separately for fruit and vegetable intake values, for example, if an outlier for fruit was removed, the non-outlier value would be retained for vegetables. A total of 176 data points for fruit or vegetable intake were excluded in UKHLS using this method. In HILDA, no intake outliers were removed. These were checked using the same method as UKHLS but because the ‘amount’ variable was limited to six pre-defined options, these were less likely than in UKHLS where a free text box was used.

Not all participants had complete parental data for various reasons (e.g. parents were not part of the original sample). There were 252 (16%) and 1932 (31%) missing values for parental education values in UKHLS and HILDA, after imputing missing values using a last observation carried forward method from previous waves. These participants were excluded only from Models 3a and 3b.

**Fig. 1:**
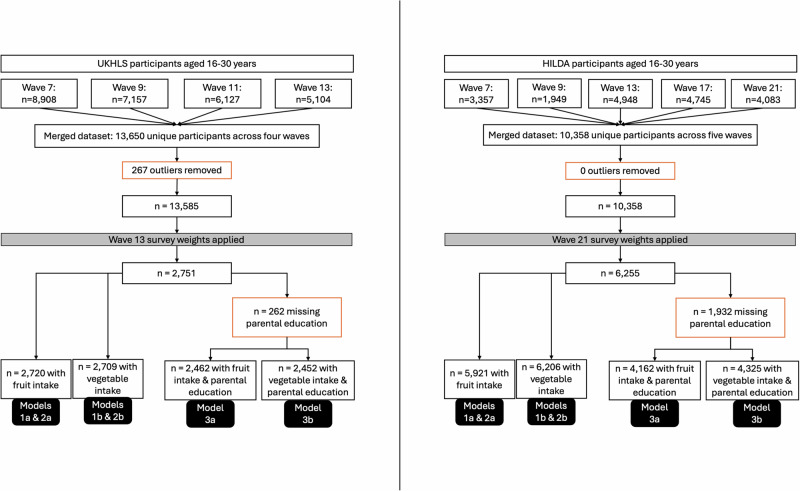
UKHLS and HILDA sample size flow charts.

Longitudinal survey weights provided by each dataset were applied according to their user manuals [43, 50]. These were based on the last wave of data included in the analysis, Wave 13 for UKHLS and Wave 21 for HILDA. This allowed for only participants who responded to each previous waves to be included in the analysis. The sample size for both datasets was reduced after applying survey weights due to some participants being assigned a weight of zero due to non-response in previous waves. Flow charts detailing sample sizes can be found in Fig. 1.

## Results

### Weighted sample characteristics and descriptive statistics

The weighted analytical sample included 2751 (UKHLS) and 6255 (HILDA) young adults aged 16–30 years. Mean fruit intake was comparable between both populations (1.42 daily portions in UKHLS and 1.32 daily portions in HILDA). Mean vegetable intake was slightly lower in UKHLS at 1.77 daily portions, compared to HILDA at 1.98 daily portions. The mean age was 20.3 years (SD: 4.7, range: 16–30 years) in UKHLS and 20.9 years (SD: 4.2, range: 16–30 years) in HILDA. Both populations had a fairly even split of female and male participants (59% and 51% females in UKHLS and HILDA, respectively). The proportion of parents with a degree was higher in UKHLS (55% vs 35% in HILDA). Table 2 shows the details of these characteristics.

### Change in fruit and vegetable intake with age (Models 1a and 1b)

In UKHLS, for each year increase in age, individual fruit intake decreased by 0.04 portions per day [95% Confidence Interval (CI): −0.05, −0.02], equating to a decrease of over a portion across a month. HILDA also showed a decrease in fruit intake of 0.01 portions per day [95% CI: −0.02, −0.01] with each year increase in age, corresponding to a 0.3 portion decrease over a month. Similarly, both populations showed a consistent trajectory in vegetable intake: as they aged, young adults increased their vegetable intake by 0.02 portions per day per year increase in age for UKHLS [95% CI: 0.01, 0.03] and HILDA [95% CI: 0.01, 0.02], equivalent to just over half a portion increase over a month. Details of all model estimates can be found in Supplementary Table S1.

### Change in fruit and vegetable intake with age by sex (Models 2a and 2b) and parental education (Models 3a and 3b)

**Fig. 2:**
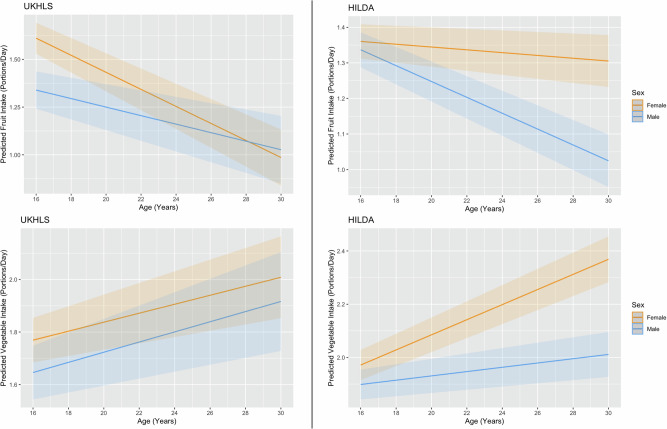
Predicted fruit and vegetable intake (portions per day) by sex for UKHLS and HILDA in young adults aged 16–30 years. Graphs include 95% confidence intervals. Note: Within-person analyses of change with age was performed using person-centred age, to reflect deviation from each individual’s mean age across all waves. In these graphs we have converted back to true age using the mean age across the cohort, to aid interpretation.

On average, in both cohorts, female participants consumed more fruit than male participants (Table S1). In UKHLS, there was a difference in intake trajectories between female participants and male participants for fruit intake only, where the difference between the groups became less apparent with age. This difference in slope was not seen for vegetable in UKHLS. In HILDA, sex moderated the relationship between age and both daily fruit and vegetable intake, with a negative interaction resulting in a more negative, or less positive, slope for male participants (β interaction = −0.02, 95% CI: −0.03, −0.01]. Figure 2 shows the contrasting trajectories of female and male participants for daily fruit and vegetable intake, where the difference in intake between the groups narrows with age for fruit only in UKHLS. The figure displays how the difference in intake trajectories between female and male participants widened with age in HILDA only, where male participants had a steeper decline in fruit intake with age than female participants, whereas, female participants had a steeper increase in vegetable intake with age than male participants.

**Fig. 3:**
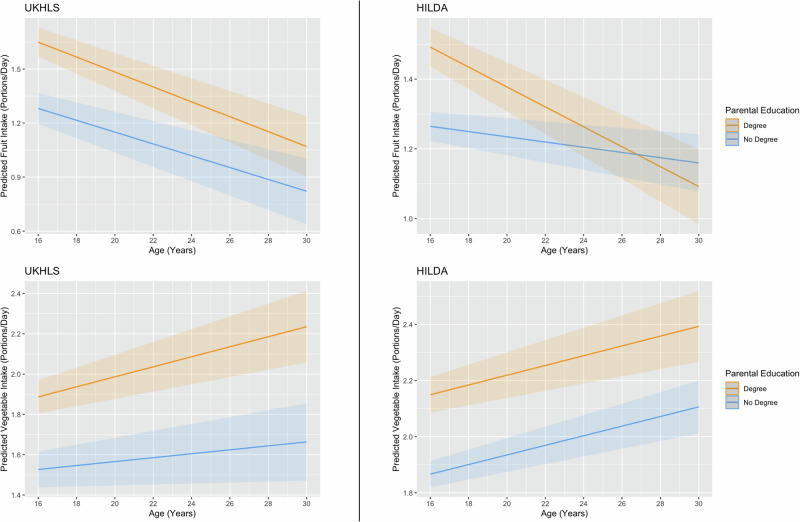
Predicted fruit and vegetable intake (portions per day) by parental education for UKHLS and HILDA in young adults aged 16–30 years. Graphs include 95% Confidence Intervals. Note: Within-person analyses of change with age was performed using person-centred age, to reflect deviation from each individual’s mean age across all waves. In these graphs we have converted back to true age using the mean age across the cohort, to aid interpretation.

In both cohorts, individuals with parents who had a degree consumed more fruit and more vegetables than those with parents educated below degree level (Table S1). Parental education moderated the relationship between age and fruit intake in HILDA only (β interaction = 0.02, 95% CI: 0.01, 0.03), where participants with parents educated to degree level and above had a steeper decrease in fruit intake with age. However, this was not the case for vegetable intake in either cohort, where the differences between groups remain consistent with age. These trajectories of intake are presented in Fig. 3.

## Discussion

### Principal findings

This is the first study to compare fruit and vegetable intake trajectories through ages 16–30 years in the UK and Australia, comparing differences by sex and SEP. Overall, female participants and individuals whose parents had a degree consumed more fruit and more vegetables in both countries. Over this age range, trajectories of fruit intake decreased with age, while trajectories of vegetable intake increased with age in both the UK and in Australia. However, the difference in intakes between female participants and male participants narrowed with age (for fruit only) in the UK and widened with age in Australia. In Australia, over a period of 10 years, the difference in both fruit and vegetable intakes between female and male participants increased by 0.2 portions each day, or six portions each month, suggesting that inequalities by sex develop over this period. Furthermore, our analysis showed the impact of parental SEP on diet, with inequalities persisting with age for vegetable intake in both cohorts and for fruit intake in the UK.

### Strengths and limitations of the study

The strengths of our study include the use of weighted, nationally representative samples and harmonized datasets, allowing for robust comparisons across the UK and Australia. The inclusion of parental-reported education enhances the reliability of the socioeconomic indicator, compared to if parental education had been reported by young adults. Our analytical approach of employing hybrid mixed effects models allowed for the disaggregation of between- and within-person effects, providing details on the effect of age on dietary intake to evaluate changes across early adulthood.

The reliance of self-reported fruit and vegetable intake and simplicity of the dietary questions introduce potential bias, with a risk of overreporting due to social desireability [51]. Although the dietary questions were comparable in both datasets, there are differing definitions of ‘portion’ in the UK and ‘serve’ in Australia, which may complicate comparisons. In Australia, a standard serve is 75 g for vegetables and 150 g for fruit and according to UK guidelines, a standard portion for both fruit and vegetables is 80 g. However, we analysed countries separately, comparing trajectories of intake rather than absolute intake, therefore, this did not affect our interpretation. Although our analysis demonstrated variations in trajectories between different sociodemographic groups, the groupings explored were limited by the comparability of available data, leaving other potentially important variables unexplored, such as geographical location, area deprivation, ethnicity or indigenous status.

The Covid-19 pandemic’s impact on dietary habits adds an additional layer of complexity to our interpretation. Shifts in fruit and vegetable intake patterns could be attributed to pandemic-related factors, as our study included data collected since March 2020. The UK experienced an increase in home food preparation time [52, 53], potentially leading to the observed increase in vegetable intake, as vegetables frequently require cooking. Qualitative findings from Australia suggested a mix of both positive and negative impacts on food preparation and consumption of the pandemic [53, 54]. However, we conducted a sensitivity analysis by excluding data points collected from March 2020 onward, and there were no significant changes to the findings (data not shown).

### Implication of findings

Young adults continue to have lower than recommended intakes of fruit and vegetables in both the UK and Australia. Our findings indicate only slight variations in intake with age, suggesting that dietary patterns established in adolescence tend to persist into early adulthood, with only minimal changes as individuals mature from age 16 to 30 years. Future research efforts could focus on trajectories through early adulthood and into late adulthood, to examine long-term dietary habits and whether these dietary patterns persist.

The observed difference in fruit and vegetable intakes between female and male participants has been previously documented [30, 31, 55, 56]. Psychosocial frameworks, such as the Theory of Planned Behaviour, suggest that women exhibit higher perceived behavioural control and more positive attitudes towards consuming fruits and vegetables, which are predictors of their higher self-reported intake [30]. To mitigate these sex-differences in intake, interventions could aim to improve men’s perceptions and attitudes towards fruits and vegetables. A healthy lifestyle intervention pilot aimed at young men aged 18–25 years in Australia demonstrated the feasibility, acceptability and potential efficacy of such interventions, with recommendations for specific recruitment, retention and research procedures to address specific motivators and barriers for this population [57]. In addition to overall sex-differences in intake, we found a widening difference with age between female and male participants in Australia but and a narrowing difference in the UK. Research on marketing and advertising has shown that the UK has implemented regulations that control harmful gender stereotyping [58], while Australia has yet to establish similar restrictions [59]. This difference in regulatory approach could help explain why our study observed a greater polarisation with age between the sexes in Australia than in the UK. As young adults age, they become increasingly exposed to and aware of gendered attitudes, which may influence their dietary choices.

The relationship between parental SEP and dietary intake examined in the present study highlights a socioeconomic divide persisting as participants aged. The observed average difference amounts to around half a portion ( ~ 40 grams) per day in combined fruit and vegetable intake between young adults from higher and lower parental SEPs. A comprehensive systematic review of observational studies has demonstrated an inverse dose-response association between fruit and vegetable intake and the risk of cardiovarscular disease-related mortality and morbidity. Specifically, an increase of 50 grams/day in fruit and vegetable intake was associated with a reduction in all-cause mortality and various disease outcomes [1]. Therefore, prioritising intiatives that not only increase intake of fruit and vegetables across all demographics, but also focus on individuals from lower SEPs to boost intake up to the same level as those from a higher SEP could significantly improve public health outcomes and reduce health inequalities. The observed disparity demonstrates the importance of parental SEP in influencing diet quality, aligning with existing literature linking parental SEP with dietary trajectories throughout life [24, 60, 61]. Childhood SEP, predicted via parental education, has been shown to be critical in predicting young adult diet quality [61, 62], with individuals in a lower SEP during childhood showing lower intakes of fruit and vegetables and higher intakes of fast food during early adulthood. These indicate the lasting effect of parental SEP on dietary habits, suggesting that a higher SEP earlier in life is conducive to healthier dietary patterns later in life. As adolescents gain independence and control over their food choices during early adulthood, they may begin to improve their diet by increasing fruit and vegetable intake. These would require considerations relating to resources available to them in terms of time, money, skills or facilities. However, if they are in a lower SEP, they may not have these resources are their disposal and would therefore struggle to increase their intake at the same rate as those that would have these resources (higher SEP individuals). The differences seen in vegetable intake as opposed to fruit is potentially due to the preparation effort involved, indicating that as young adults age, those from a higher SEP might have better opportunities to develop and use their cooking skills. The persistent gap in intake as young adults age emphasises the need for interventions tailored to address these inequalities, such as enhancing accessibility to healthy foods. For example, population level interventions such as subsidisation of healthier foods have shown to reduce inequalities, whereas person-centred approaches such as health education are more likely to widen inequalities [63]. Research suggests that the difference in diet quality among adolescents from a lower SEP may be worsened by limited availability of fruit and vegetables at home, suggesting that the home food environment also contributes to inequalities between the groups [64]. The development and maintenance of food planning skills from adolescence into early adulthood may also enhance the socioeconomic gap, as these skills are required for improved diet quality and are often influenced by parental education, making them less likely to be acquired in households of a lower SEP [65, 66]. These factors should be considered when designing and implementing interventions and policy to improve diet quality. Such measures will benefit individuals from distinct SEPs differentially, thereby having consequential impacts on health inequalities.

## Conclusion

Overall, fruit intake trajectories decreased with age and vegetable intake trajectories increased with age in 16–30 year olds in the UK and Australia. In Australia, but not in the UK, inequalities in intake of fruit and vegetables, by sex, emerged across early adulthood. In Australia, over a period of 10 years, the difference in both fruit and vegetable intakes between female and male participants increased by 0.2 portions each day, or six portions each month. Inequalities by parental education remained consistent over time for fruit and vegetables in the UK and for vegetable intake in Australia, while these inequalities narrowed over time for fruit intake in Australia. These findings highlight the need for targeted interventions and policies that not only promote increased fruit and vegetable intake among young adults but also address dietary inequalities, which could, in turn, improve long-term health outcomes and reduce the burden of diet-related chronic diseases.

## Supplementary information

Supplementary Table S1

## Data Availability

UKHLS: The data that support the findings of this study are available on request from the UK Data Service 10.5255/UKDA-SN-6614-19, study number (SN) 6614. The data are publicly available, however, they are considered safeguarded and therefore require users to register and accept the End User Licence. HILDA: This paper uses unit record data from Household, Income and Labour Dynamics in Australia Survey [HILDA] conducted by the Australian Government Department of Social Services (DSS). The findings and views reported in this paper, however, are those of the author[s] and should not be attributed to the Australian Government, DSS, or any of DSS’ contractors or partners. 10.26193/KXNEBO. The analysis code has been made available on OSF at the following link: https://osf.io/pcd97/?view_only=3ba40915dfcd467a87d57e46f8e02e37.
